# Therapeutic and Adverse Effects of a Non-Steroidal Glucocorticoid Receptor Ligand in a Mouse Model of Multiple Sclerosis

**DOI:** 10.1371/journal.pone.0008202

**Published:** 2009-12-07

**Authors:** Simone Wüst, Denise Tischner, Michael John, Jan P. Tuckermann, Christiane Menzfeld, Uwe-Karsten Hanisch, Jens van den Brandt, Fred Lühder, Holger M. Reichardt

**Affiliations:** 1 Institute for Multiple Sclerosis Research, University of Göttingen and Gemeinnützige Hertie-Stiftung, Göttingen, Germany; 2 Department of Cellular and Molecular Immunology, University of Göttingen Medical School, Göttingen, Germany; 3 Institute for Inorganic Chemistry, University of Göttingen, Göttingen, Germany; 4 Leibniz Institute for Age Research - Fritz Lipmann Institute, Jena, Germany; 5 Department of Neuropathology, University of Göttingen Medical School, Göttingen, Germany; University Paris Sud, France

## Abstract

**Background:**

Dissociating glucocorticoid receptor (GR) ligands hold great promise for treating inflammatory disorders since it is assumed that they exert beneficial activities mediated by transrepression but avoid adverse effects of GR action requiring transactivation. Here we challenged this paradigm by investigating 2-(4-acetoxyphenyl)-2-chloro-*N*-methyl-ethylammonium chloride (CpdA), a dissociating non-steroidal GR ligand, in the context of experimental autoimmune encephalomyelitis (EAE), an animal model of multiple sclerosis (MS).

**Methodology/Principal Findings:**

CpdA inhibited pro-inflammatory mediators in myelin-specific T cells and fibroblasts in a GR-dependent manner while gene activation was abolished. However, it also induced massive apoptosis in various cell types even in the absence of the GR by engaging a Bcl-2- and caspase-dependent pathway. ^1^H NMR spectroscopy corroborated these findings by revealing that CpdA dissolved in buffered solutions rapidly decomposes into aziridine intermediates known to act as alkylating pro-apoptotic agents. Importantly, the dichotomy of CpdA action also became evident *in vivo*. Administration of high-dose CpdA to mice was lethal while treatment of EAE with low to intermediate amounts of CpdA dissolved in water significantly ameliorated the disease. The beneficial effect of CpdA required expression of the GR in T cells and was achieved by down regulating LFA-1 and CD44 on peripheral Th cells and by repressing IL-17 production.

**Conclusions/Significance:**

CpdA has significant therapeutic potential although adverse effects severely compromise its application *in vivo*. Hence, non-steroidal GR ligands require careful analysis prior to their translation into new therapeutic concepts.

## Introduction

Glucocorticoids (GCs) are amongst the most potent anti-inflammatory drugs presently available and are widely used to treat chronic inflammatory diseases such as rheumatoid arthritis (RA) and multiple sclerosis (MS) [1]–[3]. Nevertheless, GC therapy is often complicated by severe adverse effects such as osteoporosis, muscle atrophy or diabetes [4], [5]. Therefore, it remains a challenge to develop new GC analogues with similar efficacy but reduced side effects.

Since their first clinical use more than half a century ago [6], GCs have been modified in multiple ways to improve their pharmacological characteristics. One strategy is based on the notion that adverse effects are often mediated by transactivation after GR binding to promoter and enhancer elements present in target genes. In contrast, transrepression was hypothesized to underlie many beneficial effects that proceed without DNA-binding and rather depend on the interference of the monomeric GR with transcription factors such as NF-κB or AP-1 [7]. Support for this concept came from of the analysis of GR^dim^ mice that are defective in GR dimerization and DNA-binding [8], [9]. Importantly, several cytokines in these mice are fully repressed by GC treatment while regulation of many genes involved in metabolic side effects is abolished [8], [9]. This model stimulated the search for new dissociating GR ligands that predominantly act via transrepression such as ZK 216348 [10], AL-438 [11] or 2-(4-acetoxyphenyl)-2-chloro-*N*-methyl-ethylammonium chloride, also known as Compound A (CpdA) [12].

CpdA is a synthetic analogue of a substance that naturally occurs in the Namibian shrub *Salsola tuberculatiformis* Botschantzev. It has contraceptive activity [12], exerts anti-androgenic effects [13] and binds to serum proteins such as corticosteroid-binding globulin (CBG) and albumin [14]. Biochemical analysis revealed that CpdA and the classical GC dexamethasone (Dex) both interact with the GR at high affinity [15]. Additionally, it could be shown that CpdA at a dose of 10^−5^ M down regulates NF-κB driven gene expression without inducing GC response element (GRE) dependent genes [15]. These results are compatible with the concept that CpdA represents a dissociating GR ligand. Analysis of two mouse models further supported the idea that CpdA has potent anti-inflammatory activity but exerts only little side effects when applied *in vivo*. Preventive administration in the acute zymosan-induced paw inflammation model in C57Bl/6 mice and therapeutic treatment of collagen type II induced RA in DBA/1 mice both efficiently interfered with disease progression in the absence of metabolic side effects [15], [16]. Therefore, CpdA was considered a promising candidate for possible future application in humans. Nevertheless, a detailed dose response analysis of CpdA *in vivo* has not been reported to date.

MS is an inflammatory demyelinating autoimmune disease of the central nervous system (CNS), which is associated with severe functional deficits. Many of its hallmarks can be studied in experimental autoimmune encephalomyelitis (EAE), a frequently used animal model for MS [17]. Initially, autoreactive T cells cross the blood-brain barrier and initiate an inflammatory response succeeded by the influx of additional leukocytes and an amplification of the immune response. High-dose GC therapy confers significant therapeutic benefit to MS patients suffering from acute relapses [3], [18], which is similarly observed after treatment of EAE. Experiments in mice revealed that T cells but not macrophages are the essential targets of GC therapy [19]. Although lymphocyte infiltration into the CNS was diminished after GC treatment, T cell apoptosis and expression of adhesion molecules in the spinal cord remained unaltered. In contrast, splenic T cells showed significantly increased apoptosis, reduced surface levels of LFA-1, CD44 and VLA-4 and impaired lymphocyte migration to the inflamed CNS [19]. Hence, GC therapy of EAE impacts T cells via the transactivating as well as the transrepressing function of the GR.

In this study, we addressed the question whether CpdA was suitable for the treatment of EAE. Although we could confirm the dissociating character of CpdA *in vitro* and *in vivo*, it additionally induced apoptosis independent of the GR in a variety of cell types including lymphocytes, neuronal cells and fibroblasts. ^1^H nuclear magnetic resonance (NMR) spectroscopy revealed that CpdA rapidly decomposes into aziridine derivatives when dissolved in buffered solutions while it is rather stable in pure water. Based on these findings we identified conditions that allowed successful treatment of EAE induced in C57Bl/6 mice. However, when CpdA was administered at high concentration it turned out to be lethal. We conclude that a detailed characterization of dissociating GR ligands is mandatory to adequately judge their suitability for future therapeutic application.

## Methods

### Mice

C57Bl/6 wildtype mice were purchased from Harlan Winkelmann (Borchen, Germany); GRN and GR^lckCre^ mice were previously described [20], [21]. All animal experiments were conducted according to ethical standards of humane animal care and approved by the authorities of Lower Saxonia (LAVES).

### Reagents

2-(4-acetoxyphenyl)-2-chloro-*N*-methyl-ethylammonium chloride (Compound A) was purchased from Merck Biosciences (Schwalbach, Germany) and stored at −80°C until use. Water-soluble dexamethasone (Dex) for cell-culture was from Sigma (Taufkirchen, Germany), dexamethasone-21-dihydrogen-phosphate for EAE therapy from Ratiopharm (Ulm, Germany) and Z-VAD-fmk from R&D Systems (Wiesbaden, Germany).

### Flow Cytometry

All antibodies and reagents were obtained from BD Biosciences (Heidelberg, Germany): anti-CD3ε (145-2C11), anti-CD4 (RM4-5), anti-CD44 (IM7), anti-CD11a/LFA-1 (2D7), anti-IFNγ (DB-1) and anti-IL-17 (TCII-18H10), 7-AAD and AnnexinV. The antibodies and AnnexinV were directly labeled with FITC, PE, PerCP, PE-Cy7, Cy5, APC or APC-Cy7. Extracellular and intracellular stainings were performed as previously described [19] and analyzed using a FACS Canto II device allowing for the detection of six fluorescent dyes (BD Biosciences).

### *In Vitro* Cell Culture Experiments

Clonal lines of encephalitogenic T (T_enc_) cells specific for guinea pig myelin basic protein (gpMBP) were established by immunization of Lewis rats followed by antigen restimulation *in vitro* as described [22]. T_enc_ cells and thymocytes were cultured in RPMI 1640 medium (Invitrogen, Karlsruhe, Germany) with Glutamax, 10% FCS and 1% standard antibiotics. Mouse embryonic fibroblasts (MEFs), WEHI 7.1 mouse thymoma and SK-N-SH neuroblastoma cells were cultured in DMEM medium (Invitrogen) with Glutamax, 10% FCS and 1% standard antibiotics.

For functional analyses, T_enc_ cells were cultured in the presence of 10 µg/ml gpMBP and irradiated T cell-depleted rat splenocytes at a 1∶1 ratio (1×10^6^ cells in total in a 24-well plate) for 12 hrs, followed by additional 5 hrs in the presence of the respective hormones. To determine cytokine production, the cells were treated with Golgi-Plug (BD Biosciences) during the last 2 hrs according to the manufacturer's instructions. To induce MMP-13 expression MEFs were treated for 5 hrs with 5 ng/ml PMA.

### Quantitative Polymerase Chain Reaction (PCR)

Total RNA was isolated using TriZol reagent according to standard procedures (Invitrogen) followed by DNaseI treatment and purification with the RNeasy Mini Kit (Qiagen, Hilden, Germany). 1 µg of RNA were reversely transcribed into cDNA using the BioRad iScript kit (Munich, Germany). Real-time PCR was performed on an ABI 7500 instrument (Applied Biosystems, Darmstadt, Germany) using the SYBR mastermix from the same company according to the manufacturer's instruction. The results were normalized to β-actin expression and evaluated using the relative quantification method.

### Generation of Retrovirally Transduced Cells

The vector pEYZ/MCS-bcl-2 is described elsewhere [23]. An shRNA specific for the mouse GR (TGCTGTTGACAGTGAGCGCG GCGATACCAGGATTCAGAAATA GTGAAGCCACAG ATG TATTTCTGAATCCTGGTATCGC CTTGCCTACTGCCTCGGA) was cloned into the retroviral vector MSCV-LMP (Open Biosystems, Huntsville, AL, USA) to obtain LMP-msiGR. Viral particles were generated following published protocols [23] and directly used to transduce WEHI 7.1 cells by spinoculation (32°C, 870 g, 3 h). Cells were selected either by culture in the presence of 2 µg/ml puromycin (LMP-msiGR) or 200 mg/ml neomycin (pEYZ/MCS-bcl-2). Purity was assessed by flow cytometry on the basis of eGFP expression and found to be greater than 99%.

### ^1^H Nuclear Magnetic Resonance (NMR) Spectroscopy

Decay of CpdA was monitored by ^1^H NMR spectroscopy at a concentration of 5 µM in 100 mM phosphate buffer with pH values of 8.1, 7.7, 7.1 and 6.7. The buffer solutions were prepared by dissolving mixtures of KH_2_PO_4_ and K_2_HPO_4_ in D_2_O followed by pH measurement using a glass electrode [24]. In addition, decay of CpdA was analyzed in pure D_2_O (pH 7.0), PBS (100 mM, pH 7.6) or Tris-HCl buffer (100 mM, pH 7.6). The dissolved CpdA was quickly transferred into the magnet of a Bruker Avance 500 MHz NMR spectrometer (Rheinstetten, Germany) using a 5 mm NMR tube followed by recording of ^1^H NMR spectra (20 scans, 2.75 s acquisition) at 25°C in intervals of 1 min for up to 3 hrs. Hydrolysis to acetyl synephrine and synephrine was monitored over a period of 21 days. Aromatic resonances were used for integration, and the peak integrals of CpdA were fitted to a monoexponential decay using Origin (OriginLab, Northampton, MA, USA). Peak assignments for CpdA, the two isomeric aziridines and acetyl synephrine were established by COSY (8 scans, 512×1024 data points, 2 h) and NOESY (8 scans, 0.5 s mixing, 2.5 h) experiments. Chemical shifts [25] and coupling constants [26] were in agreement with previous reports.

### EAE Induction and Treatment Protocols

C57Bl/6 mice or GR^lckCre^ mice on the same genetic background were immunized with 50 µg MOG_35–55_ peptide in CFA as previously described [19]. Animals were weighed and scored daily for clinical signs of the disease on a scale from 0 to 10 depending on its severity; scores were as followed: 0 = normal; 1 = reduced tone of tail; 2 = limp tail, impaired righting; 3 = absent righting; 4 = gait ataxia; 5 = mild paraparesis of hindlimbs; 6 = moderate paraparesis; 7 = severe paraparesis or paraplegia; 8 = tetraparesis; 9 = moribund; 10 = death.

To analyze the effects of CpdA therapy, the drug was dissolved in 150 µl ethanol (20% vol/vol), pure water or PBS and injected i.p. on 3 consecutive days. For comparative analysis we injected Dex dissolved in PBS following the same time schedule.

### Proliferation Assay and ELISA

Single cell suspensions were prepared from spleen of C57Bl/6 mice immunized with MOG_35–55_ on the day after the third treatment with CpdA or PBS as a control. 3×10^5^ cells were seeded in 96 well microtiter plates (Nunc, Wiesbaden, Germany) in 100 µl medium each in the presence or absence of MOG_35–55_ peptide (20 µg/ml) or ConA (1.25 µg/ml). Triplicate cultures were maintained at 37°C in a humidified incubator with 5% CO_2_ for 56 hrs and harvested following a 16 hrs pulse with 0.2 µCi/well ^3^[H]-thymidine (Perkin Elmer, Rodgau, Germany). Cells were collected on fiberglass filter paper and the incorporated radioactivity was measured using a β-plate liquid scintillation counter (Perkin Elmer). To determine cytokine secretion, 5×10^6^ cells were seeded in 24 well microtiter plates in the presence or absence of MOG_35–55_ peptide or ConA as described above. The supernatants were collected 72 hrs later and the cytokine levels were determined by commercially available ELISA kits for IFNγ (BD Bioscience) and IL-17 (R&D Systems, Wiesbaden, Germany) according to the manufacturers' instructions. All experiments were at least performed twice.

### Statistical Analysis

Analysis was performed by Mann-Whitney or Students t-test and the data depicted as mean ± SEM; *: p<0.05, **: p<0.01, ***: p<0.001, n.s.: p>0.05. To determine differences referring to the EAE disease course, the whole curves were compared between experimental groups starting on the day after the first treatment until the end of the observation period.

## Results

### CpdA Allows Dissociating GR-Mediated Transrepression from Transactivation in Myelin-Specific T Effector Cells and Mouse Embryonic Fibroblasts

**Figure 1:**
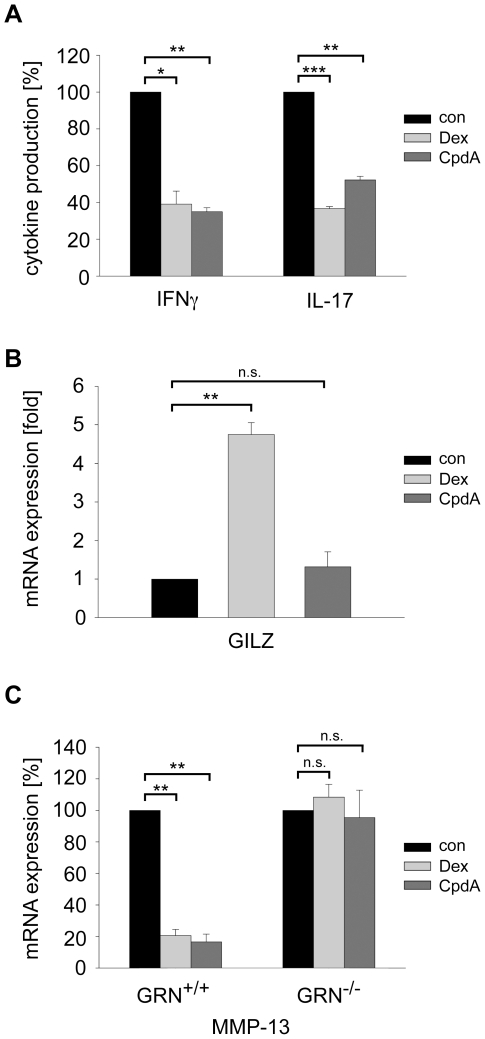
CpdA mediates transrepression in myelin-specific T effector cells via the GR but not transactivation. (A) T_enc_ cells were cultured in the presence of APCs and 10 µg/ml gpMBP, either treated with 10^−6^ M Dex or 10^−5^ M CpdA for 6 hrs or left untreated (con). IFNγ and IL-17 production was assessed by intracellular flow cytometry and normalized to each control (100%) based on the percentage of cells positively staining for the respective cytokines. n = 3. (B) T_enc_ cells were cultured in the presence of 10^−6^ M Dex or 10^−5^ M CpdA for 6 hrs followed by the analysis of GILZ mRNA expression by quantitative RT-PCR. The expression levels were normalized to the housekeeping gene β-actin and the results are depicted as fold induction relative to untreated control cells. n = 3. (C) MEFs derived from GRN^+/+^ mice (wildtype cells) and GRN^−/−^ mice (GR-deficient cells) were preincubated with 5 ng/ml PMA for 1 hr. Subsequently, they were stimulated with 10^−6^ M Dex or 10^−5^ M CpdA for 5 hrs or left untreated (con). MMP-13 mRNA expression was determined by quantitative RT-PCR and normalized to β-actin. MMP-13 levels in the medium controls were set as 100%. n = 3. *: p<0.05, **: p<0.01, ***: p<0.001, n.s.: p>0.05.

CpdA had been reported to possess properties reminiscent of *bona fide* dissociating GCs. To confirm this characteristic with regard to the suitability of CpdA for the treatment of EAE and possibly MS, we tested its transrepression and transactivation activity in encephalitogenic T cells (T_enc_). These myelin-specific T effector cells are able to induce an MS-like pathology after adoptive transfer into rodents and resemble the pathogenic T lymphocytes found in MS patients. Initially, we analyzed production of IFNγ and IL-17, two cytokines that are involved in the pathogenesis of MS [27]. Treatment of T_enc_ cells with 10^−6^ M Dex as well as 10^−5^ M CpdA for 5 hrs diminished production of both pro-inflammatory mediators by around 60% based on intracellular flow cytometric analysis (Fig. 1A). In contrast, addition of 10^−5^ M CpdA did not alter the expression of the *bona fide* GR target gene glucocorticoid-induced leucin zipper (GILZ) in T_enc_ cells while treatment with 10^−6^ M Dex strongly increased it (Fig. 1B). CpdA at a concentration of 10^−6^ M neither altered cytokine production nor GILZ expression (data not shown). Importantly, no apoptosis was detected within the 5 hrs culture period, thus excluding that the observed effects were linked to cell death (data not shown). We conclude that CpdA acts in a truly dissociating manner in myelin-specific T cells.

To examine whether CpdA exerts its repressive activity via the GR, we studied regulation of matrix metalloproteinase 13 (MMP-13) in mouse embryonic fibroblasts (MEFs). MMP-13 expression is controlled by the transcription factor AP-1 and becomes repressed by GCs through direct protein-protein-interaction of the GR with AP-1 [28]. Wildtype (GRN^+/+^) and GR-deficient (GRN^−/−^) MEFs were stimulated with PMA in the presence of 10^−6^ M Dex or 10^−5^ M CpdA followed by quantitative RT-PCR analysis. MMP-13 expression in wildtype cells was efficiently reduced by either treatment, while the inhibitory effect of both substances was fully abolished in MEFs from GRN^−/−^ mice (Fig. 1C). CpdA at a concentration of 10^−6^ M had no effect on MMP-13 expression (data not shown). Taken together, CpdA allows dissociating transactivation from GR-mediated transrepression.

### CpdA Induces Apoptosis in Various Cell Types

**Figure 2:**
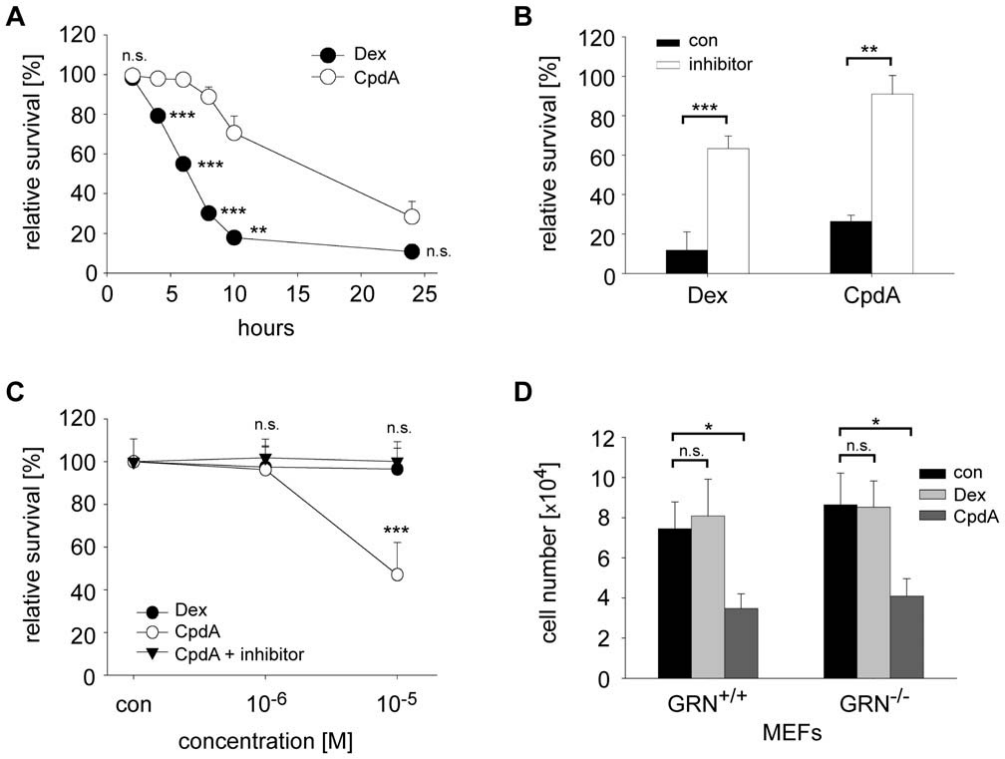
CpdA exerts pro-apoptotic activity in various cell-types. (A) Thymocytes were cultured in the presence or absence of 10^−6^ M Dex or 10^−5^ M CpdA for 24 hrs. Apoptosis was assessed at different time points by flow cytometry based on staining for AnnexinV/7-AAD. Survival of untreated cells was set as 100% for each time point to correct for spontaneous apoptosis. n = 3. (B) Thymocytes were treated with Dex or CpdA for 24 hrs as in panel A, either in the absence (con) or the presence of 100 µM Z-VAD-fmk (pan-caspase inhibitor). n = 3. (C) SK-N-SH neuroblastoma cells were treated with Dex or CpdA at concentrations of 10^−6^ M and 10^−5^ M for 24 hrs or left untreated (con). To test whether CpdA action depends on caspase-activity, the experiment was additionally performed in the presence of 100 µM Z-VAD-fmk. n = 6. (D) 5×10^4^ MEFs generated from GRN^+/+^ and GRN^−/−^ fetuses were cultured in the absence (con) or presence of 10^−6^ M Dex or 10^−5^ M CpdA. After 48 hrs cell numbers were determined by microscopic counting. n = 3. *: p<0.05, **: p<0.01, ***: p<0.001, n.s.: p>0.05.

GCs are potent inducers of lymphocyte apoptosis, which significantly contributes to their anti-inflammatory and immunosuppressive activity. In contrast, many other cell types such as neurons and fibroblasts are resistant to GC-induced cell death. To determine whether CpdA shares these features with traditional GCs, we initially cultured immature thymocytes, which are known to be particularly sensitive to GC-mediated apoptosis, in the presence of 10^−6^ M Dex or 10^−5^ M CpdA for 24 hrs. Flow cytometric analysis performed at various time intervals revealed that both substances efficiently induced thymocyte apoptosis, although with different kinetics (Fig. 2A). Eventually most cells died irrespective of the treatment, which could be prevented by incubation with the pan-caspase inhibitor Z-VAD-fmk (Fig. 2B).

To investigate potential pro-apoptotic effects of CpdA on non-hematopoietic cell types, we cultured SK-N-SH cells, a human neuroblastoma cell line, in the presence of 10^−6^ M Dex or 10^−5^ M CpdA for 24 hrs. While Dex didn't compromise the viability of these cells, incubation with CpdA strongly induced apoptosis. Again, this could be prevented by co-incubation with the pan-caspase inhibitor Z-VAD-fmk, confirming that cell death was not the result of unspecific necrosis (Fig. 2C). Thus, CpdA induces apoptosis in neuronal cells that are normally resistant to GC treatment.

In view of the unusually high apoptotic potency of CpdA we tested its effect on fibroblast growth. As expected, treatment of mouse embryonic fibroblasts (MEFs) for 48 hrs with 10^−6^ M Dex had no effect whilst CpdA significantly diminished cell numbers when added at a concentration of 10^−5^ M (Fig. 2D). This effect was mediated - at least in part - by induction of apoptosis (data not shown). Surprisingly, we obtained similar results in MEFs derived from GRN^+/+^ and GRN^−/−^ mice, indicating that the GR was not essential for the observed effects. This suggests that CpdA also engages GR-independent signaling pathways.

### CpdA Induces Apoptosis through the Mitochondrial Pathway in a GR-Independent Manner

**Figure 3:**
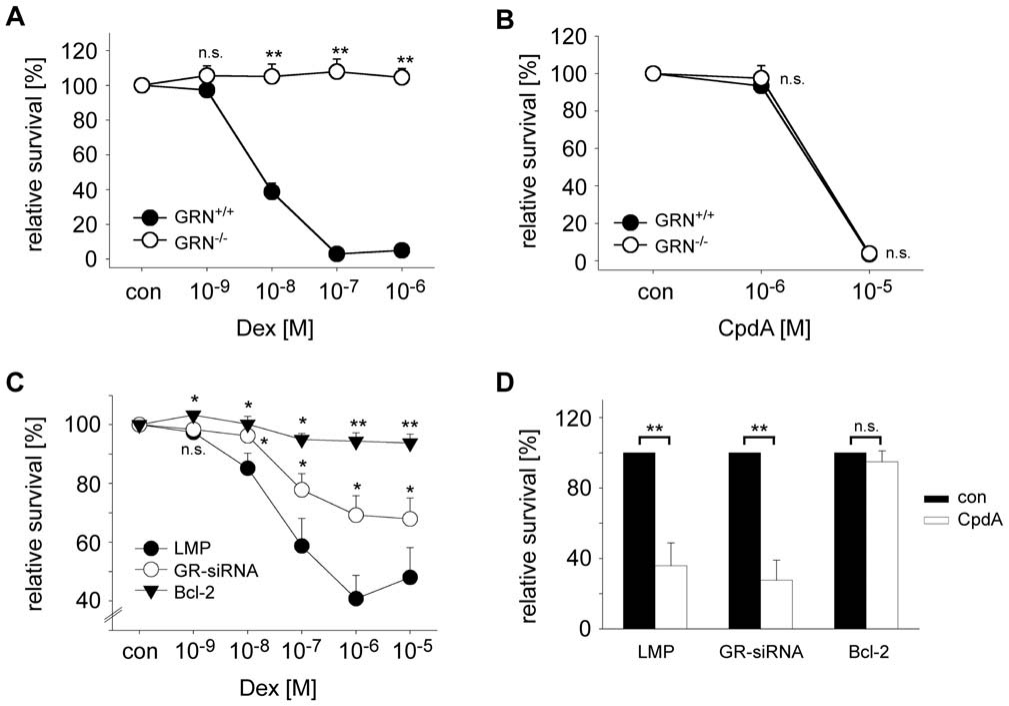
CpdA-induced apoptosis is independent of the GR. (A,B) Thymocytes were isolated either from GRN^+/+^ or GRN^−/−^ E18.5 embryos and cultured in the absence (con) or presence of different concentrations of Dex (A) or CpdA (B) for 24 hrs. Apoptosis was assessed by flow cytometry based on AnnexinV/7-AAD staining. Survival of untreated cells was set as 100% for each time point to correct for spontaneous apoptosis. n = 3. (C,D) WEHI 7.1 cells stably transduced with a retrovirus encoding a GR-specific shRNA (GR-siRNA), Bcl-2 or the empty retroviral vector LMP, were cultured in the absence (con) or presence of different Dex concentrations (C) or 10^−5^ M CpdA (D) for 24 hrs. Apoptosis was assessed by flow cytometry based on AnnexinV/7-AAD staining. Survival of untreated cells was set as 100% for each time point to correct for spontaneous apoptosis. n = 3−5. *: p<0.05, **: p<0.01, n.s.: p>0.05.

Alerted by the striking findings in MEFs we performed additional studies on the role of the GR in CpdA-induced apoptosis. Since GR-deficient mice are not viable, we analyzed thymocytes from GRN^+/+^ or GRN^−/−^ embryos at day E18.5. Incubation of wildtype cells with increasing amounts of Dex for 24 hrs resulted in the induction of cell death following a sigmoid curve [21]. In contrast, GR-deficient thymocytes were completely resistant at all Dex concentrations tested (Fig. 3A). When we treated thymocytes for 24 hrs with CpdA, cell survival sharply declined from nearly unimpaired viability at 10^−6^ M to almost complete cell death at 10^−5^ M (Fig. 3B). Most importantly, thymocytes from GRN^−/−^ embryos were as sensitive to CpdA-induced apoptosis as wildtype cells (Figure 3B). Similar results were obtained with adult T cell-specific GR knockout mice (data not shown). Collectively, CpdA efficiently induces thymocyte apoptosis in a GR-independent manner.

To obtain further insight into the molecular mechanism of CpdA, we analyzed WEHI 7.1 cells, a Dex-sensitive mouse thymoma cell line (Fig. 3C). Initially, we silenced the GR by retroviral delivery of a specific microRNA followed by incubation of the stably transduced cells with different concentrations of Dex or CpdA for 48 hrs. Flow cytometric analysis confirmed our earlier findings in thymocytes showing that GR inactivation compromised Dex-induced cell death in WEHI 7.1 cells while the pro-apoptotic activity of CpdA was unaffected (Fig. 3C,D). To explore whether the mitochondrial pathway was responsible for CpdA induced apoptosis, we expressed Bcl-2 in WEHI 7.1 cells by retroviral transduction. Interestingly, Bcl-2 overexpression completely prevented cell death caused by both drugs (Fig. 3C,D). This confirms that CpdA induces apoptosis rather than exerting unspecific toxic effects.

### CpdA Decays into Aziridine Derivatives and Synephrine in a Solvent-Dependent Manner

CpdA dissolved in phosphate-buffered solution was previously shown to decompose into the corresponding aziridine, which is inhibited by serum proteins [12]. Interestingly, such compounds encompass several highly reactive alkylating cytostatic drugs used in cancer therapy. Thiotepa for example induces apoptosis in breast cancer and leukemia cell lines through activation of the mitochondrial pathway and causes neuronal cell death, which can be prevented by pan-caspase inhibitors [29], [30]. Since CpdA exerts similar pro-apoptotic effects in a GR-independent manner, we wanted to reevaluate the conditions under which CpdA decomposes as a basis for a possible correlation between chemical decay and biological activity *in vitro* and *in vivo*.

**Figure 4:**
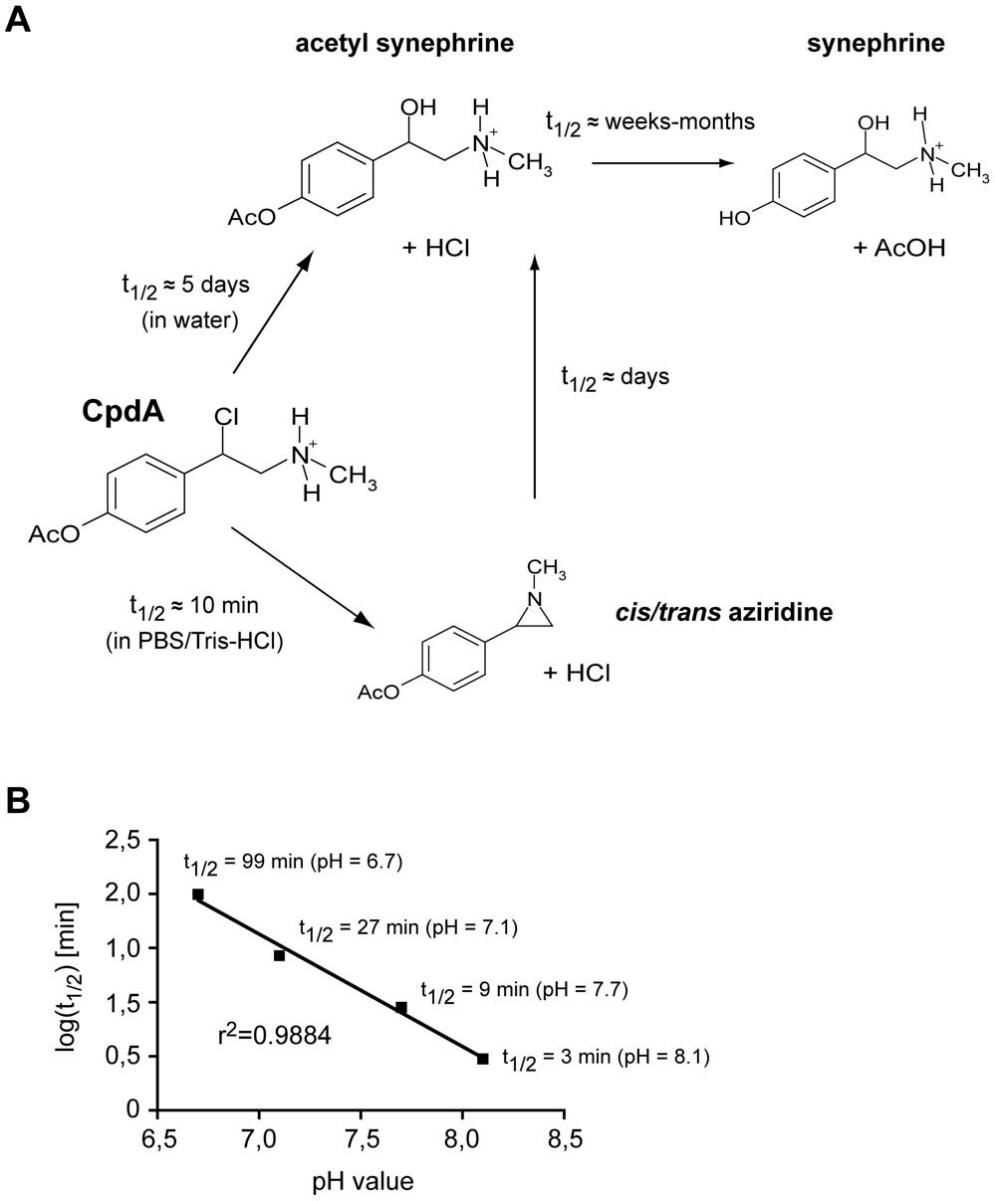
Decay of CpdA into aziridine derivatives and synephrine depends on the solvent and pH. (A) Schematic depiction of the *in vitro* decay of CpdA in pure water (upper part) and PBS (pH = 7.6) or Tris-HCl (pH = 7.6) buffer (lower part) based on ^1^H NMR spectroscopic analysis. When dissolved in water, CpdA directly decomposes into acetyl synephrine within approximately 5 days, followed by ester hydrolysis into synephrine after several weeks to months. In contrast, dissolving CpdA in buffered solutions leads to the formation of a mixture of the corresponding *cis* and *trans* aziridines, which can be detected by ^1^H NMR spectroscopy within minutes. With a half-life (t_1/2_) of several days the aziridines then become hydrolyzed into acetyl synephrine and subsequently synephrine. (B) The half-life of CpdA dissolved in phosphate-buffered solutions with pH values of 6.7, 7.1, 7.7 and 8.1 was determined by ^1^H NMR spectroscopy and its logarithm was plotted against the pH value. The exact values and the linear regression curve are depicted in the graph. r^2^ indicates the correlation coefficient.

To investigate the stability and conversion of CpdA under different physico-chemical conditions we used ^1^H NMR spectroscopy. When dissolved in pure water (pH = 7.0) CpdA slowly underwent hydrolysis to acetyl synephrine without formation of cyclic intermediates (half life: 5.5±0.3 days). Within a few weeks further hydrolysis to synephrine, an α-adrenergic receptor agonist was observed (Fig. 4A). When we dissolved CpdA in PBS (pH = 7.6) or Tris-HCl buffer (pH = 7.6) rapid formation of two reaction intermediates occurred, which we could identify as the isomeric forms of N-methyl-2-(4-acetoxyphenyl)-aziridine (Fig. 4A). Both isomers are typically formed within a few minutes, with rates 2–3 times higher in Tris-HCl as compared to PBS. Subsequently both aziridines are hydrolyzed to acetyl synephrine within a few days. Interestingly, the rate of aziridine formation is strongly pH-dependent with the half-life of CpdA varying from a few minutes at pH = 8.1 to about 2 hrs at pH = 6.7 (Figure 4B). A logarithmic plot of the half-life of CpdA versus the respective pH value showed a linear dependency with high correlation (Fig. 4B). The data are thus in agreement with the notion that cyclization - involving nucleophilic attack of the nitrogen atom under elimination of chloride - requires the amino form of CpdA, while the protonated ammonium is not reactive.

We conclude that the predominant chemical form of CpdA present in solution depends on the pH and buffer conditions. The aziridine derivatives that presumably account for the GR-independent pro-apoptotic activity are mainly formed in buffered solutions while CpdA is relatively stable when dissolved in water. Thus, the different cellular effects of CpdA could be linked to the formation of decay products.

### CpdA Exerts Dose-Dependent Therapeutic and Adverse Effects in a Mouse Model of MS

**Figure 5:**
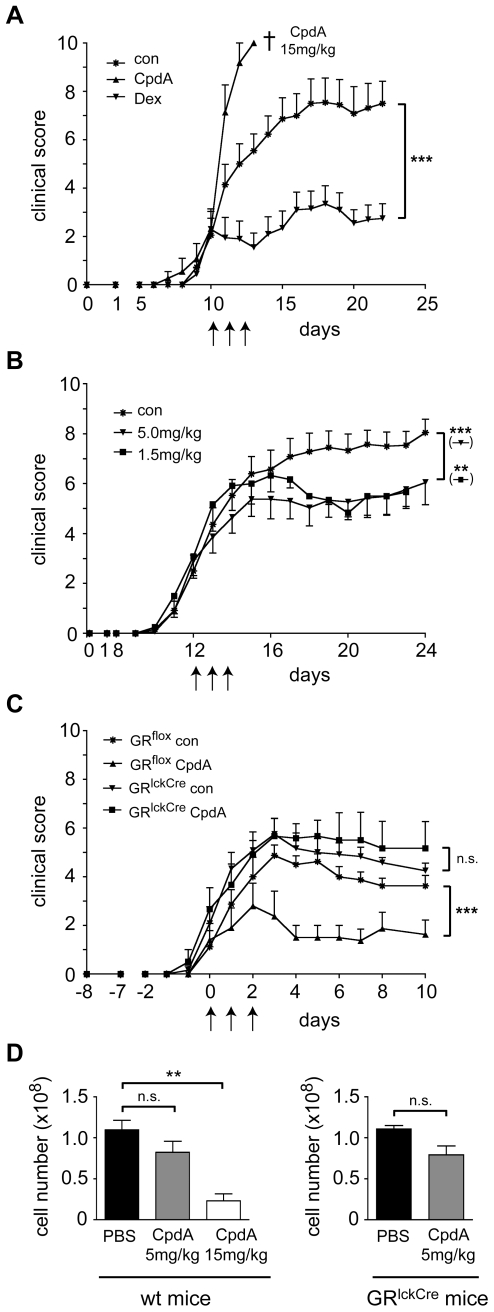
Treatment of MOG_35–55_ induced EAE by CpdA. (A) EAE was induced in C57Bl/6 mice followed by treatment with 15 mg/kg CpdA (dissolved in 20% ethanol), 100 mg/kg Dex or PBS as a control (con) on 3 consecutive days starting at an average clinical score of 2 (marked by arrows); n = 11; statistical analysis: days 11–22. The cross indicates that all animals of this group either died or had to be sacrificed for ethical reasons. The experiment was repeated twice with similar results. (B) CpdA was dissolved in water and therapeutically applied at doses of 5 mg/kg or 1.5 mg/kg for 3 consecutive days after the mice had developed an average clinical score of 3 (marked by arrows); treatment with the solvent alone (con) served as a control; n = 18/6/19; statistical analysis: days 13–23. The experiment was repeated five times with similar results. (C) EAE was induced in C57Bl/6 and GR^lckCre^ mice followed by treatment with CpdA dissolved in water at a dose of 5 mg/kg or the solvent alone (con). Therapy was started at an average score of 2 (defined as day 0); n = 6; statistical analysis: days 1–10. The experiment was repeated twice with similar results. (D) C57Bl/6 wildtype or GR^lckCre^ mice were treated with 5 mg/kg or 15 mg/kg CpdA dissolved in water or PBS as a control on 3 consecutive days. On the following morning surviving mice were sacrificed and the cellularity of the spleen was determined by microscopic counting. n = 3/12. **: p<0.01, ***: p<0.001, n.s.: p>0.05.

EAE is a widely used animal model of MS and represents one of the best-studied examples for therapeutic intervention by GCs. Since we had previously found that administration of Dex ameliorates MOG***_35–55_*** induced EAE in C57Bl/6 mice [19] and since CpdA was shown to repress inflammatory arthritis in two mouse models [15], [16], we wondered whether CpdA was also able to interfere with EAE in mice. Moreover, we were curious whether the previously observed GR-independent pro-apoptotic activity of CpdA also plays a role *in vivo* and how this may impact the suitability of CpdA as a therapeutic agent. Following published protocols we intraperitonally administered 15 mg/kg CpdA [16] or 100 mg/kg Dex [19] on 3 consecutive days to C57Bl/6 mice after manifestation of first EAE symptoms. In agreement with previous findings, Dex significantly ameliorated EAE [19]. In contrast, CpdA severely aggravated the disease course rather than having a beneficial effect. Strictly speaking, all animals died within 72 hrs after CpdA administration or had to be sacrificed for ethical reasons (Fig. 5A). Administration of 15 mg/kg CpdA to non-immunized mice was also lethal (data not shown), confirming that high-dose CpdA was intrinsically toxic. In particular, the clinical symptoms that precede death after CpdA application include abdominal pain, ataxia and intestinal necrosis at the site of injection (data not shown).

Next, we tested the therapeutic efficacy and potential side effects of CpdA at lower concentrations. In contrast to high-dose CpdA, application of 5 mg/kg as well as 1.5 mg/kg CpdA dissolved in water significantly ameliorated the disease (Fig. 5B). The beneficial effect was slightly more pronounced at the intermediate as compared to the low dose and, irrespective of its concentration, CpdA was somewhat less potent than Dex (Fig. 5A,B). Importantly, however, we didn't notice any obvious adverse effects under these conditions. Taken together, these findings suggest that treatment of EAE by CpdA is possible although only in a narrow therapeutic window. Notably, when we dissolved CpdA in PBS instead of water thereby decreasing its chemical stability (see above), the beneficial effect was partially lost (data not shown). This strongly indicates that therapeutic activity can be assigned to CpdA itself rather than the aziridine intermediate.

Finally, we determined whether the GR in T cells was essential for therapeutic efficacy of CpdA in EAE. To this end, we used conditional GR knockout mice (GR^lckCre^), which specifically lack the GR in T cells [19]. While EAE in GR^flox^ control mice could be significantly treated by 5 mg/kg CpdA, the disease course was not affected in GR^lckCre^ mice (Fig. 5C). This confirms that the therapeutic effect of CpdA is mediated via the GR by modulating T cell function.

In view of our previous finding that CpdA induces apoptosis *in vitro* at high concentrations independent of the GR (see above), we wondered whether there was a link between this feature and the side effects that CpdA exerts *in vivo*. To address this question we injected C57Bl/6 mice either with the high (15 mg/kg) or the intermediate (5 mg/kg) dose of CpdA on 3 consecutive days followed by enumerating splenocyte numbers. Treatment with the intermediate dose mildly diminished the cellularity of the spleen but without reaching significance (Fig. 5D). This effect was independent of the GR since CpdA exerted a similar effect in GR^lckCre^ mice, which are refractory to apoptosis induction by GR ligands (Fig. 5D). Remarkably, however, administration of high-dose CpdA to C57Bl/6 mice led to a strong and significant reduction in splenocyte numbers by almost 80% (Fig. 5D). We conclude that CpdA at high concentration causes massive lymphocyte death, which correlates with the manifestation of side effects.

### CpdA Ameliorates EAE by Down Regulating Cell Adhesion Molecules and Repressing IL-17 Production

Having established that CpdA - at least at certain concentrations - ameliorates EAE, we wanted to identify the underlying mechanisms. To this end, we induced EAE in C57Bl/6 wildtype mice by immunization with MOG_35–55_ peptide followed by CpdA therapy using both the intermediate and the low dose of CpdA following the same protocol as in the previous experiments.

**Figure 6:**
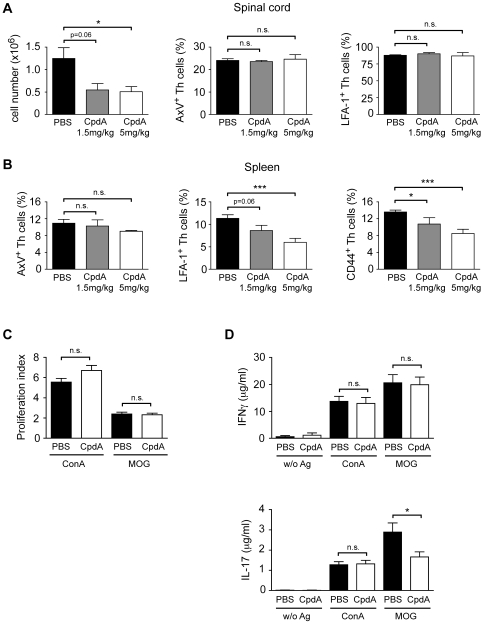
Mechanism of CpdA action in the treatment of EAE. (A) EAE was induced in C57Bl/6 mice followed by treatment with 1.5 mg/kg or 5 mg/kg CpdA dissolved in water for 3 consecutive days after the mice had developed an average clinical score of 3. On the following morning the mice were sacrificed and the leukocytes isolated from the spinal cord. Cellularity was determined by microscopic counting; staining for AnnexinV binding and surface expression of LFA-1 on CD3^+^CD4^+^ Th cells was performed by flow cytometry. n = 3−7. (B) The spleen was isolated from the same animals as in panel A and the leukocytes analyzed for AnnexinV binding as well as LFA-1 and CD44 surface expression on CD3^+^CD4^+^ Th cells by flow cytometry. n = 3−8. (C, D) Splenocytes from the same animals as in panels A and B were cultured in the presence or absence of ConA or MOG_35–55_ peptide. Proliferation was measured by ^3^[H]-thymidine incorporation assay and expressed as a proliferation index relative to the values obtained in the absence of any stimulus (C); IL-17 and IFNγ levels in the supernatant were determined by ELISA (D); n = 7−10. *: p<0.05, ***: p<0.001, n.s.: p>0.05.

CpdA treatment reduced lymphocyte infiltration into the spinal cord at 5 as well as 1.5 mg/kg, thus providing a reasonable explanation for the improved clinical disease course after therapy. However, neither the level of apoptosis nor the expression of cell adhesion molecules such as LFA-1 and CD44 were altered in CNS-infiltrating T cells (Fig. 6A and data not shown). This is in line with our previous observation that GR-mediated activities in the context of EAE are largely refined to the peripheral immune system and only indirectly impact on CNS infiltration [19]. Therefore, we subsequently studied effects of CpdA on CD4^+^ Th cells in the spleen. While the percentage of apoptotic cells was again unaltered, surface expression levels of LFA-1 and CD44 were reduced on Th cells from CpdA treated mice in a dose-dependent manner (Fig. 6B). In addition, we investigated the proliferative capacity as a measure of T cell priming and the production of effector cytokines after restimulation with ConA or the cognate antigen. There was no difference in terms of proliferation and IFNγ production between both treatment groups irrespective of whether stimulation was achieved by ConA or MOG_35–55_ peptide (Fig. 6C,D). In contrast, IL-17 levels in cultures from CpdA treated mice were significantly reduced after restimulation with MOG_35–55_, which was not the case after polyclonal stimulation with ConA (Fig. 6D). This indicates that CpdA selectively impacts antigen-specific Th17 cells known to play an essential role in the pathogenesis of EAE [31]–[33], thereby corroborating the notion that IL-17 but not IFNγ is the central mediator of this disease [34].

## Discussion

GCs are highly potent anti-inflammatory drugs that are used to treat a variety of diseases such as RA, asthma or MS [2], [3], [35]. Nevertheless, systemic application is often accompanied by unfavorable adverse effects complicating long term GC therapy [5]. Since it is believed that beneficial effects are often mediated by GR transrepression while many side effects rely on GR transactivation, current research aims at identifying novel substances that dissociate these two features. A promising candidate for such a drug is CpdA [12]. In this work we could show that CpdA indeed represses inflammatory mediators in myelin-specific T effector cells and fibroblasts *in vitro* in the absence of gene activation. However, we found that CpdA added at the same concentration also induces apoptosis in various cell-types such as lymphocytes and neuronal cells. This activity of CpdA proceeds via a Bcl-2- and caspase-dependent pathway and is fully independent of the GR. A likely explanation for the dichotomy observed in cell culture comes from our ^1^H NMR results. Despite acting in principle as a dissociating GR-ligand, CpdA dissolved in buffered solutions rapidly decomposes into aziridine derivatives that are generally known as alkylating agents with strong pro-apoptotic and neurotoxic potential [29], [30].

Benzyl halides with a β-amino group such as CpdA are known to form cyclic intermediates, which require the amino form while the protonated ammonium form is not reactive [36]. Consequently, CpdA dissolved in water is relatively stable while it rapidly decays in buffered solution with increasing pH. This brings along several biological consequences. CpdA dissolved in standard buffers such as PBS or Tris-HCl almost immediately decomposes into its aziridine derivatives and thereby induces widespread GR-independent apoptosis. Since this cannot be avoided in cell culture, massive apoptosis takes place after prolonged incubation. When CpdA is dissolved in water where it is chemically stable and subsequently administered to animals, it also decomposes into the toxic aziridines at the physiological pH of 7.4 unless quantitatively stabilized by binding to proteins such as CBG [12]. We therefore postulate that CpdA applied to mice at high concentrations results in the formation of significant amounts of the aziridine derivatives, which would explain why the mice die after injection of 15 mg/kg CpdA or have to be sacrificed for ethical reasons. Nevertheless, administration of 5 mg/kg as well as 1.5 mg/kg CpdA ameliorates EAE in a GR-dependent manner. From this we conclude that CpdA indeed possesses anti-inflammatory activity *in vivo* except that the aziridine derivatives that are presumably formed in animals at increased amounts additionally cause side effects independent of the GR. This notion is in line with our observation that low to intermediate doses of CpdA have little impact on splenic cellularity while high-dose CpdA strongly decreases leukocyte counts. We hypothesize that this toxic effect, which possibly affects also many other cell types, is responsible for the lethality observed after administration of CpdA at high concentrations.

Despite the undeniable caveats related to the *in vivo* application of CpdA, we could nevertheless confirm that it has significant therapeutic potential in the treatment of EAE if applied at a dosage of 1.5 to 5 mg/kg. Our mechanistic studies revealed that CpdA acts by down regulating expression of the cell adhesion molecules LFA-1 and CD44 on peripheral Th cells and by repressing IL-17 production by antigen-specific effector T cells. In contrast, CpdA neither has an affect on T cell priming nor on IFNγ production. This is in line with the previously proposed model that Th17 rather than Th1 cells are central to the development and pathogenesis of EAE [31], [32] as well as MS [37]. Moreover, CpdA does not induce apoptosis in a GR-dependent manner if applied at a low to intermediate dose. This corroborates the model that CpdA indeed acts as a dissociating GR-ligand *in vivo* in the treatment of EAE. In particular lymphocyte death, a *bona fide* example of GR transactivation, is not induced at these CpdA concentrations while repression of IL-17, LFA-1 and CD-44 efficiently occurs. It is noteworthy that these data are the first piece of evidence indicating that transactivation and apoptosis induction might be dispensable for the anti-inflammatory activity of GCs in the treatment of EAE and MS.

Taken together, we have confirmed the dissociating nature of CpdA *in vitro* and *in vivo* and established its therapeutic efficacy in EAE as a model of human MS if administered at certain dosages. However, we also found that it exerts GR-independent vulnerable side effects at high concentrations by inducing apoptosis *in vitro* and diminishing cell numbers *in vivo*, eventually leading to the death of all treated mice. Undeniable, this severely hampers the *in vivo* use of CpdA in humans due to the narrow therapeutic window available for the treatment of inflammatory diseases and due to the potential dramatic adverse effects that occur if CpdA is only marginally misdosed.
